# Self-rated health and its bidirectional relationship with burnout, sleep quality and somatic symptoms in a general adult population

**DOI:** 10.1186/s12889-024-19325-9

**Published:** 2024-08-02

**Authors:** Maria Nordin, Anna Sundström, Camilla Hakelind, Steven Nordin

**Affiliations:** Department of Psychology, Umeå University, Umeå, SE-901 87 Sweden

## Abstract

The aim of this study was to investigate how self-rated health (SRH) reflects ongoing ill-health and how SRH is associated with previous ill-health and/or predicts future ill-health such as burnout, disturbed sleep, and somatic symptoms. The study used two waves from the population-based Västerbotten Environmental and Health Study in which 2 336 adult persons participated by answering a questionnaire at two time points three years apart. Hierarchical and logistic regression analyses were conducted, thus treating all variables both continuously (degree) and categorically (case). The analyses were performed both cross-sectionally and longitudinally. The results showed bidirectionality between suboptimal SRH and burnout, disturbed sleep and somatic severity caseness. Moreover, degree of poor SRH was more likely to occur simultaneously to high degrees of burnout and somatic severity than to degree of poor sleep quality. Also, caseness of burnout, disturbed sleep and somatic severity increased the risk of simultaneous suboptimal SRH. Finally, the results showed that degree of burnout three years earlier, predicted degree of poor SRH, and that degree of poor SRH predicted degree of sleep three years later. In conclusion, in a population-based, normal adult sample there is a bidirectional relationship between suboptimal SRH and caseness of burnout, disturbed sleep quality and somatic symptoms, but not between degree of these symptoms. The results can have implications for health care meeting patients complaining about poor general health.

## Introduction

Self-rated health (SRH) is a holistic concept that has gained considerable attention in the past five decades for its ability to predict mortality [1] and morbidity [2]. Thanks to its general framing, the question encapsulates a variety of health domains, both mental and somatic. This quality has made the question usable across various disciplines, such as psychology, epidemiology, sociology and public health [3], and what health aspects people considers in their ratings when answering the question has been extensively studied. For example, in a study on patients in primary care, sleep problems, somatic symptoms and poor relationships were associated with suboptimal SRH [4], and in a cross-national sample aged 50 years and older, chronic conditions, difficulties moving, somatic symptoms, depression, physical activity, and education were important when rating SRH [5]. However, few studies have investigated how aspects of mental and somatic health simultaneously are related to SRH in a normal, adult population.

Understanding the implications of experiencing suboptimal health outcomes is useful for health care, not the least primary health care. With the understanding of underlying factors, more well-educated and to the point questions can be asked to disentangle various symptoms that may contribute to rating health as suboptimal by patients. It is important for the health care services to both understand whether the suboptimal health rating is a sign of ongoing ill-health or if it is related to previous ill-health. Moreover, it is of great importance to understand if feelings of suboptimal health may generate more severe ill-health in the future.

The most common cause for sick-leave in Sweden is stress-related ill-health and the prevalence keeps increasing [6]. Stress, along with sleep problems, are common underlying and consequential factors in numerous types of mental and somatic ill-health. Stress is a natural and important reaction to both external and internal stressors and includes heightened alertness and worry and hence, not so infrequently, difficulties sleeping [7]. Moreover, long-term, chronic stress is associated with emotional and physical exhaustion as well as with cognitive and somatic symptoms. Burnout is a stress-induced state, and an imbalance between stress and sleep can be a trigger for the condition [8]. In addition, disturbed sleep and burnout have been shown to have a bidirectional relationship, perpetuating each other [9].

There are different definitions of burnout. In the present study we use the conceptualisation of burnout based on Hobfoll’s Conservation of Resources (COR) Theory [10] which states that stress results from a loss of resources needed and valued. Burnout is thus a consequence of depletion of resources. It has often been studied in the workplace and is in fact defined as an occupational phenomenon in the ICD-11 [11]. However, even if the diagnostic criteria are similar to the Swedish diagnosis Exhaustion Disorder (ED) [12], with mental and physical fatigue and exhaustion in the center, ED is not limited to be due to factors occurring in the workplace but is also considered to be found in general populations [13, 14], which include both employed and unemployed people. For this study, Melamed, Kushnir and Shirom’s [15], definition of burnout is used. They concluded that burnout is a constellation of physiological fatigue, emotional exhaustion and cognitive weariness due to chronic stress related not only to work, but also to quality of life and well-being. The research on the relationship between burnout and SRH has, however, mostly been conducted on working populations. A bidirectional relationship was found between burnout and perceived health (assessed with four questions with higher values indicating better health), rated over a one-year follow-up period, with a stronger association for perceived health predicting burnout (beta = − 0.20) rather than the other way around (beta = − 0.10) [14]. Šolcová et al. [16] confirmed these results in a seven-year follow-up. As we age, our health changes, and consequently so does our ratings. Cheng et al. [17] showed in a large, representative sample, that the attribution of SRH differed between age groups; in middle-aged participants, burnout was related to SRH whereas it was attributed to disease prevalence in older participants.

Regarding sleep, Darviri et al., [18] showed the relative importance of lifestyle determinants for SRH in a cross-sectional study. Among other factors, sleep dissatisfaction in participants over the age of fifty was associated most strongly with suboptimal SRH. Interestingly, a similar association was found in adolescents, indicating an inverted U-shaped association. An inverted U-shape association was also shown in the relationship between sleep duration and SRH as sleeping both too few (less than 7) and too many (more than 9) hours was associated with suboptimal SRH [19–23]. When adding sleep quality into the equation though, SRH was rated as suboptimal only in those sleeping few hours, and the authors concluded that good sleep quality may function as a buffer against suboptimal SRH [20]. The importance of sleep quality for SRH was further confirmed in a prospective study on adolescents, showing that poor sleep quality was associated with suboptimal SRH whereas sleep duration (< 8 h per night) was not [24].

Experiencing a combination of insufficient sleep, short sleep duration and insomnia was associated with a clearly elevated risk of suboptimal SRH in a sample of female nurses. [24]. Insufficient sleep over time was also associated with suboptimal SRH in a large representative sample, thus suggesting that experiencing poor sleep over time has a cumulative effect on suboptimal SRH [25]. Moreover, poor sleep quality mediated the association between suboptimal SRH and inflammation [26].

It is not surprising that somatic symptoms such as pain, constipation, headache and a racing heart are associated with SRH since they cause discomfort and hinder health-related behavior. Pain in general has shown a stronger association to suboptimal SRH in a middle-aged sample than in an older sample [27]. Chronic pain was also independently associated with SRH in a general population. The more often pain was experienced, the higher the risk of suboptimal SRH, with daily spells of chronic pain increasing the odds of poor SRH by almost twelve times [28]. SRH predicted both incidence and persistence in pain in the arm in a general sample of adults [29] and being free of headaches was associated with better SRH together with health promoting behavior in adolescents [30]. In an older sample (> 60 years), lack of headache also increased the chance of a good SRH [31].

Stomach aches, constipation and diarrhoea are symptoms of irritable bowel syndrome (IBS), and out of several somatic diseases, SRH attributed to physical factors was most strongly associated to IBS in a study by Kutschke et al. 32. The authors also found that SRH attributed to mental factors was associated with IBS, but not with other somatic diseases such as asthma, cardiovascular diseases, diabetes, autoimmune diseases, and cancer [32]. Other somatic symptoms that can be most uncomfortable and increase the risk of suboptimal SRH is, for instance, palpitations. In fact, palpitations were found to be more robustly linked to SRH than lifestyle in a study on 40- and 42-year-olds [33]. There is emerging evidence that somatic symptoms are common in patients with burnout [34] and that disturbed sleep more often predict pain than the other way around [35], which indicates an intricate interaction among somatic and mental symptoms.

Taken together, there is ample evidence for the association between SRH and both mental and somatic health. However, few studies on this topic have been based on samples from the general population. Furthermore, there is a lack of literature taking the basic health concepts of stress, sleep, and somatic symptoms simultaneously into account. However, Mildestvedt et al. [4] included sleep and somatic symptoms in a cross-sectional study on SRH in primary care patients. They found that both sleep problems and somatic symptoms were associated with suboptimal SRH, indicating co-morbidity. Stress, sleep and somatic symptoms have strong connections with each other and are often co-morbid states [8, 34]. However, they may also appear independent of each other.

To study the relationships between the concepts of stress, sleep and somatic symptoms in SRH, this study aims to investigate how SRH reflects ongoing ill-health and to examine if SRH is associated with previous ill-health and/or predicts future ill-health, operationalized as burnout, sleep, and somatic symptoms.

## Methods

The relationships between the concepts of burnout, sleep and somatic symptoms in SRH were studied by using two waves, collected three years apart, from the population-based Västerbotten Environmental Health Study (VEHS); by studying both *degree* and *caseness* of the three health conditions and by studying the concepts individually and together.

### Population and sample

VEHS is a prospective, population-based survey. The aim of VEHS is to study various aspects of mental and somatic health but has a special focus on symptoms associated with environmental factors in the normal population. Since stress, sleep and somatic symptoms are common factors in ill-health, validated psychometric instruments were included in the questionnaire sent to a sample of 8 520 adult individuals (18 to 79 years old) in the county of Västerbotten which is located in northern Sweden. Västerbotten has approximately 270 000 inhabitants, and an age and sex distribution similar to that of the general Swedish population. The sample that was invited to participate in the baseline data collection in 2010 (T1), was stratified on age and sex. The invitation contained a questionnaire together with information about the aim of the study, research ethics and informed consent. A reminder was sent to non-responders after three full weeks. An additional reminder and a new copy of the questionnaire were sent after yet another three weeks. Out of the 8 520 invited individuals, 3 406 (40.0%) participated. At follow-up, in 2013 (T2), 2 336 (68.5% of the 3 406 that responded in T1) participated. Thus, 27.4% of the invited sample participated in both data collections. All questionnaires were responded to between March and May to avoid the impact of seasonal allergy, the dark period and midnight sunlight. The participants are described on background variables in Table 1.

**Table 1:** Description of sample and differences between those reporting good and suboptimal self-rated health by chi-square tests and t-test.

	Self-rated health			
	**Good** *n* = 1 706	**Suboptimal ** *n* = 630	p	
Age; m (SD)	51.94 (15.59)	59.91 (13.39)	< 0.001	
Women; n (%)	951 (55.7)	355 (56.3)	0.81	
University education; n (%)	349 (20.7)	245 (39.3)	< 0.001	
Married or co-habitant; n (%)	1337 (78.9)	460 (73.5)	< 0.05	
Living alone; n (%)	257 (15.1)	144 (23.1)	< 0.001	
Physical activity ≥ 2 times/week; n (%)	482 (28.3)	235 (37.3)	< 0.001	
Somatic symptoms (PHQ-15); n (%)	204 (11.9)	258 (40.1)	< 0.001	
Disturbed sleep quality (KSQ); n (%)	351 (20.6)	261 (41.4)	< 0.001	
Burnout (SMBQ); n (%)	163 (9.5)	200 (31.7)	< 0.001	
*Physician-based lifetime diagnoses*				
Hypertension; n (%)	351 (20.6)	276 (43.8)	< 0.001	
Rheumatism; n (%)	35 (2.1)	71 (11.3)	< 0.001	
Back/joint/muscle disorder; n (%)	148 (8.7)	208 (33.0)	< 0.001	
Migraine; n (%)	58 (3.4)	38 (6.0)	< 0.01	
Fibromyalgia; n (%)	14 (0.8)	43 (6.8)	< 0.001	
Irritable bowel syndrome; n (%)	20 (1.2)	33 (5.2)	< 0.001	
Allergic asthma; n (%)	61 (3.6)	46 (7.3)	< 0.001	
Non-allergic asthma; n (%)	55 (3.2)	34 (5.4)	< 0.05	
Post-traumatic stress disorder; n (%)	7 (0.4)	9 (1.4)	< 0.05	
Generalized anxiety disorder; n (%)	2 (0.1)	16 (2.5)	< 0.001	
Chronic fatigue syndrome; n (%)	2 (0.1)	13 (2.1)	< 0.001	
Depression; n (%)	52 (3.0)	57 (7.5)	< 0.001	
Stress-induced exhaustion disorder; n (%)	51 (3.0)	51 (8.1)	< 0.001	

PHQ-15 = 15-item Patient Health Questionnaire (score ≥ 10); KSQ = Karolinska Sleep Questionnaire (disturbed quality ≥ 3 times/week); SMBQ = Shirom Melamed Burnout Questionnaire (score ≥ 4.0)

### Measurements

#### Demographic variables

The questionnaire sent to the sample included both background questions, single-item measures as well as psychometric instruments. As for demographic variables, data on age, sex, marital status, living conditions, education and physical activity were collected, and considered as confounding variables. Age was assessed on a continuous scale and sex was categorized as woman = 1 and man = 2. Marital status was indicated by choosing from the response options *married, unmarried, divorced or widow/widower*, and participants who responded yes on the question *Do you live alone?* were identified as living alone. High education was categorized as *university/college studies* and conducting *physical activity* at least two times per week or more was considered as being physically active. Moreover, information on diseases that had been diagnosed by a physician at any point in life, was collected by asking the participants respond to a checklist of diagnoses.

#### Self-rated health

SRH was measured by using the single item question *In general*, *how would you rate your health?* [36]. The response options were (1) *excellent, (2) very good, (3) good, (4) fair* and (5) *poor*. Thus, high scores represent poor SRH. The variable was used in its original form, using all scale steps for the variable *degree of poor SRH*. By combining the response options excellent, very good and good, participants with good SRH were identified (coded as 0) and by combining the remaining two options, participants were categorized with s*uboptimal SRH* (coded as 1).

#### Burnout

To assess burnout, the Shirom-Melamed Burnout Questionnaire (SMBQ) [37, 38] was used. The SMBQ consists of twenty-two items, rated on a seven-point scale ranging from (1), *never or almost never* to (7), *always or almost always.* In this study we calculated a global score as the mean of all the twenty-two items (max score 7) and refer to this mean score as *degree of burnout*. *Burnout caseness* was identified as those with a score above 4.0 [39, 40] and the variable is coded 0 for scores below 4.0 and 1 for scores above. The SMBQ has good construct validity and reliability [37]. The internal consistency in our sample, measured by Cronbach’s alpha, was 0.95 at both T1 and T2.

#### Sleep

The Karolinska Sleep Questionnaire (KSQ) [39] was used to assess *sleep quality*. The KSQ asks whether the participants have had various sleep-related symptoms in the past three months: (0) *never*, (1) *seldom (occasionally)*, (2) *sometimes (several times per month)*, (3) *often (1–2 times per week)*, (4) *most of the times (3–4 times per week*), and (5) *always (5 times or more per week).* The variable *degree of poor sleep quality* was constructed by calculating the mean of the items *difficulty falling asleep, repeated awakenings with difficulty going back to sleep, premature awakening and disturbed sleep, difficulties waking up, not being well rested on awakening*, and *feeling of exhaustion at the awakening*. We also created a dichotomous *disturbed sleep quality*-variable by keeping in accordance with the DSM-5 criteria of insomnia and identifying the participants who had responded with the option 4 or above on at least one of the items (i.e., having some kind of disturbed sleep quality at least three times per week for the past three months). The disturbed sleep quality variable was coded with 0 for no disturbed sleep and 1 for disturbed sleep. The KSQ has good reliability, construct validity, and criterion validity [41]. The internal consistency in the present data set was good as Cronbach’s alpha was 0.83 at T1 and 0.84 T2. To assess *sleep duration*, we included a question on how many hours the participants estimated they slept on average per night.

#### Somatic symptoms

The Patient Health Questionnaire 15 (PHQ-15) was used to assess somatic symptoms. This scale includes the fifteen most common somatic symptoms (e.g., *stomach pain, headaches*, and *shortness of breath*) reported in out-patient settings [42]. The PHQ-15 regards the extent to which the participants are bothered by the various somatic symptoms. The response options are (0) *not bothered at all*, (1) *bothered a little and* (2) *bothered a lot*. Menstrual cramps is one of the items in the PHQ-15. Scores therefore range from 0 to 28 for men and between 0 and 30 for women, with higher scores representing more somatic symptoms. Due to the total score being higher for women than for men, the percentage of total score is used [41]. A mean score was calculated from these percentages and referred to as *degree of somatic symptoms* in this study. Kroenke, Spitzer, and Williams [42] identified three cut-off scores for the PHQ-15, 5, 10 and 15, and exceeding 10 symptoms is being considered as problematic. Hence this value was set as cut-off score when creating the variable *case of * s*omatic severity*. Due to the sex differences in total score, the recommended cut-off score [42, 43] was converted to percentage (33.3%) of total score, meaning a cut-off of 10 + for women, and 9 + for men. PHQ-15 has shown adequate internal consistency [42, 44] and moderate convergent validity [43]. For the present sample, the internal consistency was 0.81 at T1 and 0.80 at T2.

### Statistical analysis

Missing values were estimated with multiple imputation, using fully conditional Markov chain Monte Carlo methods with ten maximum iterations through which five imputed datasets were created, and values were averaged across these datasets. The percentage missing values were 0% for SRH, 2.3% for SMBQ, 2.0% for KSQ, 1.3% and 4.4% for PHQ-15. T-tests and chi-square tests were used to examine differences in background variables between participants with good and suboptimal SRH.

Pearson’s correlation coefficients were calculated to investigate the cross-sectional associations between scores on burnout, sleep quality, somatic symptoms and SRH, separately at T1 and T2. The scores were also used to study the relationships between degree of poor SRH, on the one hand, and degree of burnout, poor sleep quality and somatic symptoms, on the other hand, by performing hierarchical regression analyses. We studied six relationships, and to check for linearity we conducted polynomial regression analyses. As it turned out, only two of these relationships met the criteria for linearity (degree of burnout at T1 predicting degree of poor SRH at T2, and degree of poor SRH at T1 predicting degree of sleep quality at T2). The other four relationships (degree of poor sleep quality at T1 predicting degree of poor SRH at T2; degree of somatic severity at T1 predicting degree SRH at T2; degree of poor SRH at T1 predicting degree of somatic severity at T2; and degree of poor SRH at T1 predicting degree of burnout at T2) met the criteria for second order relationships. Therefore, for the four latter relationships, the independent variables were centred around their means respectively and squared before entered in the hierarchical regression analyses.

The analyses were performed cross-sectionally and longitudinally and conducted in blocks. In the first cross-sectional analyses we used degree of poor SRH at T1 as the independent variable. The first block included age, sex, education, marital status, living conditions and physical activity at T1. In block 2 we added degree of burnout, poor sleep quality, and somatic symptoms at T1 simultaneously in order to take the holistic perspective into account. Reverse analyses were conducted with degree of burnout, poor sleep quality, and somatic symptoms at T1 as independent variables. The first block, again, included the demographic variables age, sex, education, marital status, living conditions and physical activity at T1. In the second block degree of poor SRH at T1 was included.

The longitudinal analyses were set up in a similar way with (i) degree of poor SRH at T2 as the dependent variable, regressed by degree of burnout, poor sleep quality and somatic symptoms at T1, and, to test for reversed associations, (ii; reversed causation) degree of poor SRH at T1 as the independent variable predicting degree of burnout, poor sleep quality and somatic symptoms at T2. In analysis (i), the first block included age, sex, education, marital status, living conditions and physical activity at T1, block 2 included degree of poor SRH at T1 (to account for the within-subject design), and in block 3 degree of burnout, poor sleep quality and somatic symptoms at T1 were added. In analysis (ii), the first block included age, sex, education, marital status, living conditions and physical activity at T1, block 2 included degree of burnout, poor sleep quality and somatic symptoms at T1 (to account for the within-subject design), and block 3 included degree of poor SRH at T1. No multicollinearity was found in any of the hierarchical regression analyses since all VIF values ranged between 1 and 2. A VIF value exceeding 10 can be problematic and indicate too high correlations between the variables [45].

To predict caseness, binary logistic regression analyses were used. We built these statistical models in analogue with the hierarchical regressions analyses, but with suboptimal SRH at T2 as the dependent variable and case of burnout, disturbed sleep quality and somatic symptoms at T1 as independent variables. For reversed associations, cases of burnout, disturbed sleep quality and somatic symptom at T2 were used as dependent variables, and suboptimal SRH at T1 as the independent variable. In the first step in both analyses, we controlled for baseline measures of the dependent variables (to account for the within-subject design). In the second step we used the same demographic variables as in the hierarchical analyses as confounding variables. In the last step we added the independent variables at T1. To keep the statistical models as parsimonious as possible, a thorough confounding control was conducted by correlating the potential confounding variables with the independent variables and testing for causality with the dependent variables by regression analyses. Moreover, interaction analyses were conducted to evaluate potential moderating effects of age. Due to the number of analyses conducted, the alpha-level was set at 0.01 to decrease the risk of type 1 error. IBM SPSS versions 27 and 28 [46, 47] were used for all analyses.

## Results

### Study sample

Of the total sample, 1 706 (73.0%) participants reported excellent, very good or good SRH at T1. At T2, three years later, these levels were very similar with 1 725 (73.8%) reporting excellent, very good or good health. The groups are further described in Table 1. Compared to those with good SRH, participants with suboptimal SRH were older. Moreover, a larger proportion had university education, were living alone, met the criteria for physical activity, experienced somatic symptoms, disturbed sleep quality and burnout, and reported each of the thirteen diagnoses given by a physician. A smaller proportion of the participants with suboptimal SRH were married or co-habitants. Regarding the sex distribution, the groups did not differ significantly.

The mean score for burnout was 2.57 (SD = 0.98) in the group with good SRH, and 3.47 (SD = 1.19) in the group with suboptimal SRH (t (df = 959.24) 16.98; *p* < .001). The mean score for sleep quality was 1.34 (SD = 0.77) for the group with good SRH, and 1.85 (SD = 0.93) for the group with suboptimal SRH (t (df = 967.75) 11.99; *p* < .001). There was no difference between the groups regarding sleep duration (mean = 7.05 h, SD = 1.00 vs. 7.04, SD = 1.38 respectively; t (df = 868.20) 0.08; *p* = .47). For the group with good SRH, the mean somatic symptom score was 5.03 (SD = 3.86) and for the group with suboptimal SRH the corresponding score was 9.17 (SD = 5.10), displaying significantly different scores  (t (df = 1234) 17.96; *p* < .001).

#### Cross-sectional results

Table 2 shows Pearson’s correlation coefficients between degree of poor SRH, burnout, poor sleep quality and somatic symptoms at T1 (lower part) and T2 (upper part). Positive and significant correlations of moderate strength were found between degree of poor SRH and burnout and somatic symptoms, at both T1 and T2 (*r* = .45 − .57). Positive correlations of weak strength were found between degree of poor SRH and poor sleep quality (*r* = .24), at both T1 and T2.

**Table 2:** Cross-sectional correlation coefficients between degree of dependent and independent variables at T1 (lower part) and T2 (upper part).

T2	Poor SRH	Burnout	Poor Sleep Quality	Somatic Symptoms
T1				
Poor SRH		.48***	.24***	.49***
Burnout	.45***		.42***	.57***
Poor Sleep Quality	.24***	.42***		.35***
Somatic Symptoms	.47***	.54***	.37***	

**p < .01 ***p < .001

To investigate how SRH reflects ongoing ill-health, cross-sectional analyses were conducted. The results from the cross-sectional hierarchical analyses performed at T1 are shown in the upper part of Table 3. The first block ,which included confounding variables, explained 12%. The second block, including degree of burnout, poor sleep quality and somatic symptoms explained most of the variance, 21%. The results show that the higher the degree of burnout and somatic symptoms, the higher was the degree of poor SRH (Table 3 upper part). Similarly, the higher the degree of poor SRH, the higher was the degree of burnout (when taking sleep and somatic symptoms into account) and somatic symptoms (when taking burnout and sleep into account) (Table 4, upper part). However, degree of poor sleep quality did not seem to play a big role in degree of SRH in a cross-sectional perspective.

**Table 3:** Cross-sectional and longitudinal results from hierarchical regression analyses predicting degree of poor self-rated health (SRH).

	Model change			Standardized coefficients	
	∆ R^2^	∆F	p	β	p
**Cross-sectional results at T1**					
Block 1:	0.12	63.67	< 0.001		
Age				0.17	< 0.001
Marital status				0.04	< 0.05
Living conditions				− 0.05	< 0.05
Education				− 0.18	< 0.001
Physical activity				0.19	< 0.001
Block 2:	0.21	238.40	< 0.001		
Burnout				0.44	< 0.001
Poor sleep quality				0.00	0.84
Somatic symptoms				0.07	< 0.01
**Longitudinal results**					
Block 1:	0.10	50.79	< 0.001		
Age				0.17	< 0.001
Marital status				0.04	< 0.05
Living conditions				− 0.01	0.52
Education				− 0.18	< 0.001
Physical activity				0.12	< 0.001
Block 2:	0.37	1573.99	< 0.001		
Poor SRH at T1				0.65	< 0.001
Block 3:	0.01	16.58	< 0.001		
Burnout				0.13	< 0.001
Poor sleep quality				− 0.03	0.13
Somatic symptoms				0.01	0.77

**Table 4:** Cross-sectional and longitudinal results from hierarchical regression analyses on degree of self-rated health (SRH), burnout, sleep quality and somatic symptoms.

	Burnout					Sleep quality					Somatic symptoms				
	Model change			Stand coeff		Model change			Stand coeff		Model change			Stand coeff	
	∆ R^2^	∆ F	p	β	p	∆ R^2^	∆ F	p	β	p	∆ R^2^	∆ F	p	β	p
**Cross-sectional results T1**															
Block 1:	0.04	29.47	< 0.001			0.01	4.44	< 0.01			0.01	16.26	< 0.001		
Sex				− 0.10	< 0.001				− 0.06	< 0.05					
Age				− 0.08	< 0.001										
Marital status									− 0.04	0.07				0.45	0.10
Education														− 0.11	< 0.05
Physical activity				0.14	< 0.001				0.02	0.44					
Block 2:	0.10	131.37	< 0.001			0.11	146.41	< 0.001			0.10	124.50	< 0.001		
Burnout									22.	< 0.001				0.17	< 0.001
Sleep quality				0.22	< 0.001									0.22	< 0.001
Somatic symptoms				0.18	< 0.001				0.21	< 0.05					
Block 3:	0.17	569.45	< 0.001			0.00	0.19	0.74			0.00	18.37	< 0.01		
SRH				0.45	< 0.001				0.01	0.77				0.10	< 0.001
**Longitudinal results**															
Block 1:	0.01	10.98	< 0.001			0.03	21.84	< 0.001			0.01	12.43	< 0.001		
Sex				− 0.10	< 0.001				− 0.15	< 0.001					
Age				− 0.05	< 0.05										
Marital status									0.04	< 0.05				0.07	< 0.01
Education														− 0.08	< 0.01
Physical activity				0.02	0.80				− 0.07	< 0.01					
Block 2:	0.09	77.79	< 0.001			0.21	212.43	< 0.001			0.15	113.71	< 0.001		
Burnout T1				0.17	< 0.001				0.42	< 0.001				0.07	< 0.01
Sleep quality T1				0.15	< 0.001				0.13	< 0.001				0.07	< 0.05
Somatic symptoms T1				0.09	< 0.01				− 0.01	0.53				0.34	< 0.01
Block 3:	0.00	0.55	0.46			0.01	43.17	< 0.001			0.00	5.12	< 0.05		
SRH				0.02	0.46				0.14	< 0.001				0.51	< 0.05

Note: The models include the confounding variables found relevant to each model

### Predicting SRH

To understand whether SRH reflects previous ill-health or predicts future health problems, we conducted longitudinal analyses. Results from the longitudinal hierarchical regression analyses predicting degree of poor SRH at T2 are shown in the lower part of Table 3. Block 1, including demographic variables, explained 10% of the variance, while degree of poor SRH at T1 explained most of the variance, 37% in block 2. Entering degree of burnout, poor sleep quality and somatic symptoms in block 3 added only 1% of the explained variance. Moreover, the results showed that the higher the degree of burnout, the higher was the degree of poor SRH, whereas the associations for somatic symptoms and poor sleep quality were non-significant.

The longitudinal results showed that the higher the degree of burnout, the higher was the degree of poor SRH three years later. However, neither degree of somatic symptoms nor degree of poor sleep quality predicted SRH at T2.

Results from the binary logistic regression analyses predicting suboptimal SRH are presented in Table 5. Caseness of burnout, disturbed sleep quality, and somatic severity independently predicted suboptimal SRH at T2 three years later, with somatic severity being the strongest predictor (OR = 2.28). To check for independency of the predictors, burnout, disturbed sleep quality and somatic symptom caseness were added in each model suitable (see Table 5). The ORs for each predictor remained significantly larger than unity when the other predictors were entered in the model, indicating that they all do predict suboptimal SRH independently.

**Table 5:** Three-year prediction of self-rated health (SRH) from burnout and disturbed sleep quality caseness as well as having more than ten somatic symptoms. Results from binary logistic regression analyses.

	Suboptimal SRH at T2		
**T1**	OR	CI	p
Burnout case^1^	1.62	1.21–2.17	< 0.01
Burnout case^2^	2.04	1.50–2.77	< 0.001
Burnout case^2^ + disturbed sleep quality	1.61	1.16–2.23	< 0.01
Burnout case^2^ + somatic severity	1.64	1.19–2.25	< 0.01
Burnout case^2^ + disturbed sleep quality & somatic severity	1.43	1.03-2.00	< 0.05
Disturbed sleep quality ^1^	1.70	1.33–2.17	< 0.001
Disturbed sleep quality ^2^	1.93	1.50–2.50	< 0.001
Disturbed sleep quality ^2^ + burnout case	1.67	1.27–2.20	< 0.001
Disturbed sleep quality ^2^ + somatic severity	1.61	1.22–2.11	< 0.001
Disturbed sleep quality ^2^ + somatic severity & burnout case	1.47	1.10–1.95	< 0.01
Somatic severity ^1^	2.28	1.75–2.91	< 0.001
Somatic severity ^2^	2.35	1.79–3.09	< 0.001
Somatic severity ^2^ + burnout case	2.09	1.57–2.77	< 0.001
Somatic severity ^2^ + disturbed sleep quality	2.05	1.54–2.73	< 0.001
Somatic severity ^2^ + disturbed sleep quality & burnout case	1.92	1.44–2.57	< 0.001

^1^Adjusted for SRH at T1.

^2^Additionally adjusted for age, marital status, living conditions, education and physical activity at T1.

### Predicting burnout, poor sleep quality and somatic symptoms

To further understand the relationship between SRH and future health, SRH was used as the independent variable. The results from the longitudinal hierarchical analyses predicting burnout, poor sleep quality and somatic symptoms at T2 are shown in Table 4 (lower part). The demographic variables entered in block 1 explained 1% of degree in burnout, 3% of degree in sleep quality and 1% of degree of somatic symptoms at T2 (Table 4). Entering block 2 revealed that degree of burnout, poor sleep quality and somatic symptoms at T1 explained most of the variance in degree of burnout, poor sleep quality and somatic symptoms at T2 (9–25%) whereas degree of poor SRH explained 1% of the variance in degree of poor sleep quality and 0% in degree of burnout and somatic symptoms at T2 when entered as the third block. Degree of poor SRH predicted degree of poor sleep quality (when taking burnout and somatic symptoms into account), but neither somatic symptoms nor burnout at T2 (Table 4, lower part; alpha set at 0.01).

Results from the logistic regression analyses showed that suboptimal SRH at T1 predicted cases of both burnout, disturbed sleep quality and somatic symptoms severity (see Table 6). The OR was highest in the model predicting somatic severity and lowest in the model predicting disturbed sleep quality. Taken together, the results on caseness show a bidirectional relationship.

**Table 6:** Results from binary logistic regression analyses of three-year prediction of cases of burnout, disturbed sleep quality (T2) from self-rated health (SRH) (T1).

T2	Burnout			Disturbed sleep quality			Somatic severity		
**T1**	OR	CI	P	OR	CI	p	OR	CI	p
Suboptimal SRH^1^	3.75	2.99–4.71	< 0.001	2.60	2.14–3.17	< 0.001	2.96	2.29–3.84	< 0.001
Suboptimal SRH^2^	2.75	2.09–3.62	< 0.001	1.97	1.59–2.45	< 0.001	2.70	2.10–3.47	< 0.001
Suboptimal SRH^2^ + DSQ	2.57	1.95–3.39	< 0.001				2.37	1.82–3.07	< 0.001
Suboptimal SRH^2^ + SSS	2.33	1.75–3.10	< 0.001	1.76	1.41–2.22	< 0.001			
Suboptimal SRH^2^ + BC				1.76	1.40–2.21	< 0.001	2.55	1.97–3.29	< 0.001
Suboptimal SRH^2^ + DSQ & SSS	2.26	1.70–3.01	< 0.001						
Suboptimal SRH^2^ + BC & SSS				1.64	1.29–2.06	< 0.001			
Suboptimal SRH^2^ + BC & DSQ							2.32	1.79–3.03	< 0.001

BC = Burnout case, DSQ = Disturbed sleep quality, SSS = Somatic symptoms severity.

^1^Adjusted for baseline values for each outcome respectively.

^2^Burnout model additionally adjusted for age, sex and physical activity at T1. Disturbed sleep quality model additionally adjusted for sex, marital status and physical activity at T1. Somatic severity model additionally adjusted for marital status and education at T1.

It is noteworthy that some ORs for the adjusted models increased compared to the crude models. Scrutinizing the impact of the confounding variables showed that age was the variable that elevated the ORs the most. We therefore suspected that age could be a moderating factor, especially in the relationship between burnout caseness and suboptimal SRH. Consequently, we conducted interaction analyses with age but could rule it out as a moderating factor as the results were not statistically significant.

## Discussion

This prospective study set out to investigate the relationships between of the concepts stress (operationalized as burnout), sleep and somatic symptoms in SRH. The results show bidirectionality regarding caseness, but not regarding degree of these symptoms. More specifically, the results show that the higher the degree of burnout and somatic severity, the poorer is SRH, and vice versa. Not surprisingly, suffering from caseness of burnout, disturbed sleep quality and somatic severity increase the risk of reporting suboptimal health. However, whereas sleep does not seem to play a big role in SRH in the cross-sectional perspective, it seems to do so in the long run. Poor SRH seems to be indicative of poorer sleep and somatic severity (but not burnout) three years later. However, only burnout seems to lead to poorer SRH according to our results. Also, caseness of burnout, disturbed sleep quality and somatic symptoms were all independently related to suboptimal SRH. Somatic symptoms, followed by burnout, showed the strongest predictive relationships with suboptimal SRH, indicating that experiencing somatic symptoms and mental exhaustion would be the most important factors for reporting suboptimal SRH.

It is worth noting that the explained variance in the hierarchical regression analyses did not change when entering burnout, sleep quality and somatic symptoms as predictors of self-rated health at T2 after having included the variable SRH at T1 into the model (Table 3, lower part), or when including SRH as predictor for sleep quality and somatic severity at T2 after having entered the sleep quality and somatic severity at T1 into the model (Table 4, lower part. This may indicate that the variance is shared between these concepts. This, in turn, may be explained by the fact that there often is comorbidity and covariance between stress, sleep and somatic symptoms and that these often co-occur in ED (8,35,49). Moreover, there may be conceptual overlaps between the psychometric instruments used. For instance, the PHQ-15 includes a question on low energy and one on sleep problems, which may have diluted the results for somatic symptoms and somatic severity and underestimated them. Nevertheless, the results showed no multicollinearity between the studied variables and attempts to disentangling the different concepts from each other may be a useful approach for the health care. For instance, our results point to the fact that it is more likely that a patient who complains about suboptimal health also have a higher degree of burnout and somatic symptoms rather than problems sleeping. But even so, caution must be taken when interpretating the studied variables’ independent association to SRH.

Being a case and consequently experiencing problems with burnout, disturbed sleep quality or severe somatic symptoms, predicts suboptimal SRH three years later (ORs 1.43–1.92) in our study. Interestingly, the odds for contracting burnout, disturbed sleep quality or severe somatic symptoms increase even more when reporting suboptimal SRH three years earlier (ORs 1.64–2.32). Thus, the ORs are higher for the direction that SRH predicts ill-health even if we cannot establish any statistically proven differences between the two temporal associations. Anyway, our results corroborate the temporal direction found by Vinokur et al. [48] on military personnel. They found that the direction was stronger for perceived health predicting burnout than the other way around and their results were further confirmed by Šolcová et al. [16] in a seven-year follow up in 45-year-olds.

In the present study, no association was found between SRH and sleep duration. Similar results were reported by Conklin et al. [23] whereas other studies have shown associations between SRH and hours slept per night [19–22]. However, these studies also found that sleep quality was more strongly associated to SRH than was sleep duration, and even though the associations between SRH and sleep quality were the weakest in our study, it was clear that there was a relationship. The association between disturbed sleep quality and suboptimal SRH was also shown in a study by Darviri et al., [18] on both adolescents and older participants (> 50 years).

Suboptimal SRH increased the risk of reporting disturbed sleep quality at T2 three years later in our study. Not feeling healthy, as reflected in suboptimal SRH, can cause worry and there is a strong relationship between worrying and disturbed sleep. In fact, worry can lead to a vicious circle of disturbed sleep and, in turn, lead to an insomnia diagnosis [49–51].

Caseness of somatic severity had a stronger relationship to SRH than disturbed sleep. Even though our study is conducted in a population-based sample, the results are in line with studies using clinical samples. Mildestvedt et al. [4], showed that suboptimal SRH was associated with somatic health complaints to a larger extent (OR 2.31; 95% confidence interval 2.03–2.67) than sleep problem (OR 1.81; 95% confidence interval 1.60–2.01) in a general, clinical sample. The fact that comparable results were found in population-based versus clinical samples corroborate the results that having severe somatic symptoms is more strongly related to reporting poor health than is disturbed sleep.

PHQ-15 covers many different bodily symptoms, such as pain, constipation, headache and heart racing, and several studies have confirmed an association between such symptoms and SRH [28–30;33,34]. Sensations from the body is a strong factor linked to the perception of one’s health. In addition, the perception of not feeling healthy may manifest itself in the body. Palmer et al. [29] showed, for instance, that belief about pain in the arm increased when attributing it to stress, exemplifying the interaction between somatic symptoms and stress. Other evidence for the close relationship between somatic symptoms and stress were shown by Glise et al. [34]. They found that somatic symptoms are common in patients with burnout which was confirmed in persons with high levels of burnout in the general population by Hammarström et al. [52]. Moreover, the relationship between burnout and disturbed sleep has previously been established [53, 54]. Also, an emerging literature mapping the association between somatic symptoms, such as pain, and sleep, suggests disturbed sleep to be a larger predictor of pain than the other way around [16]. The findings on the interplay between mental and somatic health, may explain the fact that even if burnout, disturbed sleep quality and severity of somatic symptoms were independent predictors of SRH, they also explained parts of each other’s increase in risk.

The positive correlations in the cross-sectional analyses indicate that degree of SRH, burnout, sleep quality and somatic symptoms are present simultaneously. The attempt to bring a holistic perspective in this study by including both mental and somatic factors, should be contrasted with more detailed investigations to further increase the understanding of the underlying fundamental aspects of SRH. The studies of Šolcová et al. [16] and Coombe et al. [54] highlight the importance of sub-dimensions of burnout and sleep in SRH. Our results could thus be followed-up by scrutinizing the different sub scales in both the SMBQ and KSQ as well as the items in the PHQ-15.

Age has played a moderating role in the association between sleep, somatic symptoms and SRH in many studies [17, 18, 27, 31]. The results from our study did, however, not corroborate these findings. Sex has also previously been shown to be of importance for SRH [55] but played no significant role in our data. An explanation for this may be that being set in a population-based sample, the concepts burnout, disturbed sleep quality and somatic symptoms would be distributed over all ages and sexes. Also, even if more women than men are diagnosed with for instance ED, men suffer from the symptoms to a comparable degree [34]. Moreover, headaches, shortness of breath (due to, for instance, asthmatic problems) and stomach-related problems are found both in young and old persons [43] as is poor sleep [39].

The focus of this study was to investigate the relationship between SRH and burnout, sleep and somatic symptoms. However, the perception of health is of course dependent on other factors as well, such as for example disease. It is apparent from our results, that more participants with diagnoses given by a physician report suboptimal SRH compared to participants with good SRH. This could be both due to the direct effects from the diagnoses themselves, and by their symptoms that may prevent activity and decrease quality of life. It could also be due to stress and worry and loss of sleep over the disease, which in turn could end up in suboptimal health. This is a very interesting question to study further, but not possible with our data since the question on life-time diagnoses does not state for how long the participants have had their diagnosis. Some may have recuperated and not be bothered by them anymore. This highlights the importance of the framing of questions. The time framing is not only relevant to the question on diseases, but also to the psychometric instruments used in this study. Both the PHQ-15 and SMBQ ask about symptoms during the last month, and KSQ about symptoms during the last three months. The question about SRH is, however, framed to be assessed right here and now. Only one day of stress or pain, or one night of poor sleep, may for sure affect mood and thereby affect the ratings, although in order for general health (which is what SRH regards) to be affected, symptoms may have had to occurred for a while. Thus, since the PHQ-15, SMBQ and KSQ ask for symptoms retrospectively, they may have some predictive value on general SRH in the present, in and of themselves, also in a cross-sectional statistical model.

### Strengths and limitations

This large-scale study is a population-based, prospective study reflecting a normal population. It also uses well-validated psychometric instruments assessing both the basics in health and ill-health and a holistic perspective. These factors are clear strengths of the study. A limitation though is its low response rate at T1 (40.0%), although the rate was considerably higher at T2 (68.5%).

Also, the fact that the questionnaire had a focus on symptoms associated with environmental factors may have deterred people who did not find this relevant to their own lives, from responding. This may have resulted in selection bias with consequences for representativeness. However, it has been shown that participation rates between 30 and 70% are at most weakly associated with bias [56]. An additional limitation may be the three-year follow-up period which may have been too long to detect certain relationships.

### Conclusion and practical implications

Suffering from ill-health, such as caseness of burnout, disturbed sleep and somatic severity was found to increase the risk of reporting suboptimal health. Also, suboptimal health increased the risk of future somatic severity, burnout and disturbed sleep caseness. Taken together, these results show a bidirectional relationship between suboptimal health and both mental and somatic ill-health.

Our results also show that it is more likely to rate health to be poor due to higher degree of burnout or bodily symptoms than degree of poor sleep. Moreover, degree of burnout three years earlier seems to predict poor SRH, whereas poor SRH seems to predict poor sleep three years later.

These results can be helpful in health care practice by checking primarily for ongoing symptoms of burnout and sensations from the body when a patient complains about poor health. The results also show the importance to check for mental and somatic problems (such as manifest burnout, disturbed sleep and somatic symptoms) and to take the time perspective into account, especially since suboptimal health may be an indication of developing more serious health issues later on.

## Data Availability

Data is available upon request.
